# Clinical indicators of bacterial meningitis among neonates and young infants in rural Kenya

**DOI:** 10.1186/1471-2334-11-301

**Published:** 2011-11-01

**Authors:** Michael K Mwaniki, Alison W Talbert, Patricia Njuguna, Mike English, Eugene Were, Brett S Lowe, Charles R Newton, James A Berkley

**Affiliations:** 1 Centre for Geographic Medicine Research (coast), Kenya Medical Research Institute, PO Box 230, Kilifi, Kenya; 2 Department of Paediatrics, University of Oxford, The John Radcliffe Hospital, Oxford OX3 9DU, UK; 3 Department of Psychiatry, University of Oxford, OX3, UK; 4 Centre for Clinical Vaccinology and Tropical Medicine, University of Oxford, Churchill Hospital, Oxford, OX3 7LJ, UK

**Keywords:** meningitis, "young infants", neonates, "lumbar puncture", "clinical signs", "resource-poor"

## Abstract

**Background:**

Meningitis is notoriously difficult to diagnose in infancy because its clinical features are non-specific. World Health Organization (WHO) guidelines suggest several indicative signs, based on limited data. We aimed to identify indicators of bacterial meningitis in young infants in Kenya, and compared their performance to the WHO guidelines. We also examined the feasibility of developing a scoring system for meningitis.

**Methods:**

We studied all admissions aged < 60 days to Kilifi District Hospital, 2001 through 2005. We evaluated clinical indicators against microbiological findings using likelihood ratios. We prospectively validated our findings 2006 through 2007.

**Results:**

We studied 2,411 and 1,512 young infants during the derivation and validation periods respectively. During derivation, 31/1,031 (3.0%) neonates aged < 7 days and 67/1,380 (4.8%) young infants aged 7-59 days (p < 0.001) had meningitis. 90% of cases could be diagnosed macroscopically (turbidity) or by microscopic leukocyte counting. Independent indicators of meningitis were: fever, convulsions, irritability, bulging fontanel and temperature ≥ 39°C. Areas under the receiver operating characteristic curve in the validation period were 0.62 [95%CI: 0.49-0.75] age < 7 days and 0.76 [95%CI: 0.68-0.85] thereafter (P = 0.07), and using the WHO signs, 0.50 [95%CI 0.35-0.65] age < 7 days and 0.82 [95%CI: 0.75-0.89] thereafter (P = 0.0001). The number needed to LP to identify one case was 21 [95%CI: 15-35] for our signs, and 28 [95%CI: 18-61] for WHO signs. With a scoring system, a cut-off of ≥ 1 sign offered the best compromise on sensitivity and specificity.

**Conclusion:**

Simple clinical signs at admission identify two thirds of meningitis cases in neonates and young infants. Lumbar puncture is essential to diagnosis and avoidance of unnecessary treatment, and is worthwhile without CSF biochemistry or bacterial culture. The signs of Meningitis suggested by the WHO perform poorly in the first week of life. A scoring system for meningitis in this age group is not helpful.

## Background

Meningitis, pneumonia and sepsis in neonates and young infants (age < 60 days) are leading causes of childhood death in developing countries [1,2]. These conditions have usually been studied collectively as 'serious bacterial infections'. Penicillin and gentamicin, are recommended by the World Health Organization (WHO) where any of these three conditions are suspected [3]. Meningitis is notoriously difficult to distinguish clinically in this age group because its features may be non-specific. In Bangladesh, [4] more than a quarter of neonates with suspected sepsis; but without clinical signs of meningitis, had CSF findings suggestive of meningitis. The recognition of meningitis is important given the high mortality and neuro-developmental sequelae of the disease. The latter, may be higher in case of a missed diagnosis or partial/inadequate duration of treatment when an infant is managed for the less specific condition 'neonatal sepsis' [5-7].

The WHO guidelines list 'specific' (convulsions, bulging fontanel) and 'general' (lethargy, coma, reduced feeding, irritability, abnormal cry, apnoeic episodes) signs, advising lumbar puncture (LP) if any are present [3]. These guidelines are based on evidence from the WHO multicentre study of the aetiology of serious bacterial infections in young infants in low-income settings, and expert opinion [8-10]. Independent predictors of serious bacterial infection identified in the multicentre study included feeding difficulty, lack of spontaneous movement, fever, agitation, lower chest wall indrawing, tachypnoea, grunting, cyanosis, convulsions, bulging fontanel and slow capillary refill [10]. A reduced set of signs performed as well as those from the WHO study in predicting severe disease in young infants [11]. However, in all these analyses, meningitis was grouped with bacteraemia, radiological diagnosed pneumonia, hypoxemia and death as 'severe disease'. Furthermore, the WHO multicentre study included only 33 cases of confirmed meningitis in infants < 60 days old, with only six in the first week of life. Hence, current guidelines for LP or presumptive treatment for meningitis among neonates and young infants in developing countries are based on limited data. Furthermore, LP is an under-used investigation among children in Kenyan hospitals  [12]. This is partly because of the uncertainty of interpretation without full biochemical and microbiological analysis of CSF, which is lacking in most hospitals in the region.

We have previously identified clinical indicators of bacterial meningitis in children aged 60 days or more at our hospital in rural Kenya [13] and the findings have been incorporated into Kenyan national policy [13,14]. Here we report findings using similar methods among 2,411 young infants age < 60 days during a four-year period validated using 1,512 young infant admissions over the subsequent two years, and examine the 'specific' and 'general' signs suggested by WHO [3]. We also determined the proportion of cases of meningitis that could be diagnosed without CSF biochemistry or bacterial culture and calculated the number of LPs needed to be performed to identify one case of meningitis using our indicators and those suggested by WHO. Finally we examined the feasibility of developing a scoring system using the identified clinical indicators to determine need for LP and or presumptive treatment for acute bacterial meningitis.

## Methods

### Location and patients

We conducted the study at Kilifi District Hospital, Kenya. We prospectively collected data on all admissions to identify clinical indicators of meningitis from June 1^st ^2001 to July 31^st ^2005, and subsequently to validate the findings from January 1^st ^2006 to December 31^st ^2007. The hospital is located in a rural area on the Kenyan coast serving a population of ~240,000 who are mainly rural farmers. Malaria is endemic and children receive up to 120 infective mosquito bites per year. The government of Kenya introduced Infant vaccination against *Haemophilus influenzae *type b in December 2001. HIV infection is present in 6-8% of women attending the hospital antenatal clinic. There is little antibiotic pre-treatment before admission to hospital.

### Procedures

We collected a standardized set of clinical and laboratory data (including a complete blood count and blood culture) from all young infants at admission [15]. Clinical officers and junior doctors trained in the recognition of standardized clinical signs recorded their findings directly on a computer database at admission before any results from blood tests or LP were available. Clinical management protocols followed national and WHO recommendations [3,14]. Consent for use of the data was obtained from the guardian of every child at the point of admission. The Kenyan National Ethical Review Committee approved the study.

The indications for LP were any suspicion of meningitis or sepsis; impaired consciousness, inability to breastfeed or convulsions. We delayed LP if cardiac or respiratory compromise was present. Following admission, the clinical team reviewed all admissions at least once daily and performed an LP if they subsequently suspected meningitis. Neonates and young infants with suspected invasive bacterial infection were treated with benzyl penicillin (50,000 units/kg every 6-12 hours, depending on age), plus gentamicin (3-7.5 mg/kg once daily depending on age and weight) as recommended by WHO [3]. Antimicrobial therapy, including increased dosing with penicillin for meningitis, was guided by laboratory findings and clinical response.

Cerebrospinal fluid (CSF) leukocyte count was determined with a modified Neubauer counting chamber. A Gram stain and latex agglutination antigen testing for *Haemophilus influenzae *type b and *Streptococcus pneumoniae *(Murex Diagnostics, UK) were performed if the CSF leukocyte count was > 10 cells/μl [16]. CSF was cultured using standard techniques: 20 μl of CSF was inoculated onto plates of 7% horse-blood agar and 5% chocolate agar and incubated at 36·5°C for 18-24 h. We further incubated plates without visible signs of growth for additional 24 h. We discarded plates without growth at this stage. We processed blood cultures using a BACTEC^® ^9050 system instrument (Becton Dickinson, New Jersey, USA). Pathogens in CSF or blood culture were identified by standard techniques [15,17].

**Figure 1:**
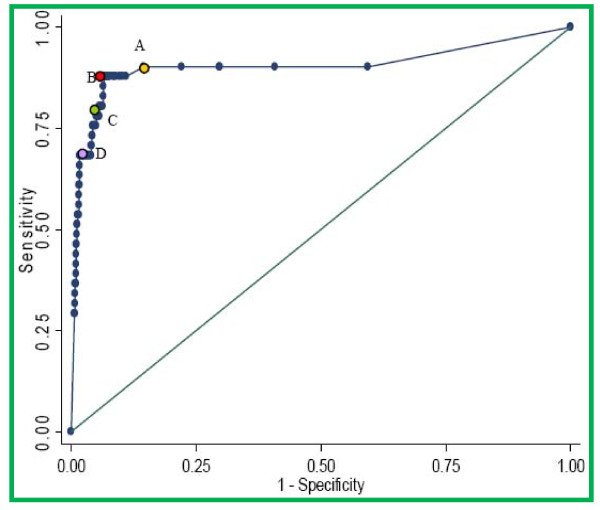
**Receiver operating characteristic curve of CSF total leukocyte count as a predictor of microbiologically confirmed meningitis in Kenyan neonates and young infants**. A = 10 leukocytes per μl, B = 50 leukocytes per μl, C = 100 leukocytes per μl, D = 500 leukocytes per μl.

For this analysis, we defined meningitis as a positive CSF culture, or a positive CSF latex agglutination test, or bacteria seen on Gram stain, or a CSF total leukocyte count ≥ 50 cells/μl. Since the cut-off of ≥ 50 cells/μl was previously derived for children age ≥ 60 days, [16] we first confirmed its validity among neonates and young infants by constructing a receiver operating characteristic curve (ROC) for total CSF leukocyte count versus CSF culture positivity (Figure 1). The area under the curve was 0.90 [95%CI 0.83-0.97]. The point of maximum discrimination was at 54 leukocytes/μl. The positive predictive value (PPV) of ≥ 50 cells/μl for a positive culture was 15% [95%CI 9.4-20.0], negative predictive value (NPV) 99.7% [95%CI 99.5-99.9]. Where CSF culture was negative but the CSF leukocyte count was ≥ 50 cells/μl and the blood culture was positive, we regarded the bacterial species isolated from blood culture as the organism causing meningitis.

### Statistical analysis

We excluded from the analysis infants who died before an LP was performed, because we could not be certain of their meningitis status. We classified those in whom we did not perform an LP, but were discharged home well as not having meningitis. We analysed data from neonates in the first week of life separately because we hypothesized that both the aetiology and the clinical signs may differ. We investigated the diagnostic values of individual clinical features by examining their positive and negative likelihood ratios (LR) for meningitis. Variables found to have crude LRs of ≥ 1.5 or ≤0.67 were regarded as potentially useful and were adjusted for the potential confounding effects of related variables in a multivariate analysis according to the method of Spiegelhalter and Knill-Jones [13,18,19]. We regarded as independent clinical indicators and retained variables with adjusted likelihood ratios (aLR) of ≥ 1.5 or ≤0.67

We derived practical screening rules from the indicators identified in descending strength of their adjusted positive LR. We then evaluated these rules by calculating the area under the ROC curves for the derivation and validation data sets. In order to examine the feasibility of developing a scoring system for meningitis, we constructed simplified scores by assigning points approximating to the natural logarithm of the adjusted likelihood ratio for each indicator. Where potential for a negative score existed, we added a constant. We also evaluated these scores by calculating the area under the ROC curves for the derivation and validation data sets. In addition, ROC curves were used to determine the cut off value with the best sensitivity and specificity in discriminating between those with or without meningitis. The negative predictive value (NPV) and the positive predictive value (PPV) were also calculated.

Before undertaking the analysis, we used the method of Hanley and McNeil to determine that the validation dataset was sufficiently large to reject a null hypothesis of no prediction (ROC area = 0.5), for areas of ≥ 0.6 [20]. ROC areas were compared using the STATA command *roccomp *for independent datasets, which returns a chi-squared statistic [21]. For other comparisons, we compared proportions using the chi-squared test with Fishers exact test where appropriate. We finally calculated the number of LPs we needed to perform to identify one case of meningitis using our signs and those suggested by WHO. We did this by subtracting the risk of meningitis in the group with indicator(s) of interest from that of the group without indicators of interest in order to obtain the absolute risk difference/reduction. The inverse of this value is the number of LPs we needed to perform to indentify a single case of meningitis [22]. We performed all analysis using STATA 9.2 (Stata Corp. USA).

## Results

**Figure 2:**
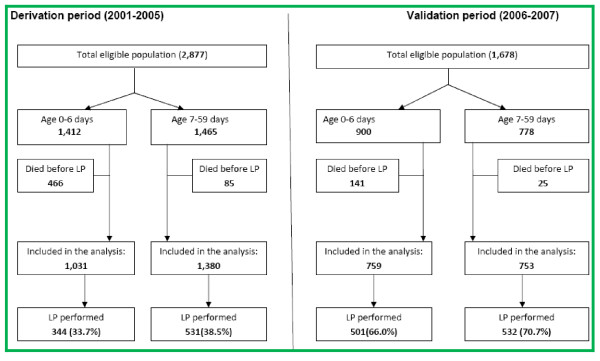
**Flow chart of study participants**.

Of 2,877 young infants admitted in the derivation period, 1,415 (49%) were age < 7 days and 1,462 (51%) were aged 7-59 days. 2,360 (82%) were delivered at home. Among those admitted in the first week of life, half were of low weight: 269 (19%) weighed < 1.5 Kg and 427 (31%) were between 1.5 and 2.5 Kg. Among infants who died, an LP was not performed in 381/447 (85%) infants admitted in the first week of life and 85/128 (66%) infants aged 7 to 59 days. Most deaths occurred within 48 hours and 40% of all deaths were among neonates admitted on the first day of life. Amongst those weighing < 1.5 Kg, overall case fatality was 63%, mainly due to prematurity and birth asphyxia. Thus, 466 neonates and young infants died before an LP was performed and were excluded from the analysis, leaving 2,411 neonates and young infants: 1,031 (43%) in the first week of life and 1,380 (57%) aged 7 to 59 days. The flow chart of patients included in the study and the number having an LP is as shown (Figure 2).

Meningitis was diagnosed less frequently in the first week of life than at 7-59 days: 31 (3.0%) vs 67 (4.8%) (p < 0.001). The commonest bacterial species were *Streptococcus pneumoniae, Group B Streptococcus and Haemophilus influenzae *(table 1). Overall, 27/98 (28%) meningitis cases could have been diagnosed by visual inspection of a turbid CSF and a further 61 (62%) by microscopic CSF leukocyte counting. A positive blood culture was found in 70/98 (71%) cases of meningitis. The case fatality ratios for young infants with meningitis were 8/31 (26%) in the first week of life and 12/67 (18%) after the first week (P = 0.37).

**Table 1:** Bacterial species found in Kenyan neonates and young infants with meningitis.

	Derivation period		Validation period		
	Firstweek of life	Age 7 to 59 days	First week of life	Age 7 to59 days	Total
**Gram positive**					
Streptococcus pneumoniae	1	10	0	5	16
Group B Streptococci **	2	5	0	6	13
Group A Streptococci	0	2	0	3	5
Group D Streptococci	1	0	1	0	2
*Streptococcus viridans*	0	0	1	1	2
*Staphylococcus aureus*	1	0	0	1	2
Gram positive cocci*	1	1	0	1	3
Gram positive rods	0	0	2	2	4
*Clostridium difficile*	0	0	0	1	1
**Gram negative**					
Haemophilus influenzae	1	6	0	1	8
Enterobacter sp.	3	2	0	0	5
Non-typhoidal Salmonella sp.	0	3	0	4	7
Escherichia coli **	0	2	1	0	3
Pseudomonas aeruginosa **	1	1	0	0	2
*Psedomonas cepacea*	0	0	0	1	0
*Klebsiella pneumoniae*	1	1	0	1	3
Acinetobacter sp.	1	1	0	0	2
Aeromonas sp.	1	0	0	0	1
Kluyvera sp.	1	0	0	0	1
*Vibrio cholera*	1	0	0	0	1
Neisseria meningitidis	0	1	0	0	1
Shigella sp.	0	1	0	0	1
Gram negative rods*	1	1	0	0	2
					
No organism isolated***	15	31	10	21	77
**Total**	**32**	**68**	**15**	**48**	**163**

* seen on Gram stain, but blood and CSF cultures and antigen tests were negative.

** One child grew *E. coli *and Group B Streptococci and another grew *Pseudomonas aeruginosa *and Acinetobacter sp.

in CSF the total is therefore 54 isolates from 98 cases of meningitis in the derivative period.

*** diagnosis based on CSF leukocyte count (see text).

Amongst infants in the first week of life, 316/1,031(31%) had a history of fever including 24/31 (77%) of those with meningitis (table 2). The crude LRs for history of fever or convulsions (partial or generalised) suggested that these might be useful indicators. The absence of a history of fever was strongly associated with the absence of meningitis. Of examination findings, agitation, bulging fontanel, and axillary temperature were the only variables to have crude LRs suggestive of predictive value (table 3). 12/31 (38.7%) cases on meningitis had the specific signs of convulsion or bulging fontanel. Place of delivery (home or hospital) was not significantly associated with meningitis.

**Table 2:** Crude likelihood ratios for meningitis for items of clinical history amongst neonates and young infants in the derivative period.

			First week of life				Age 7 to 59 days		
	Sample size		Without meningitis	With meningitis	Crude LR	Samplesize	Without meningitis	With meningitis	Crude LR
**Fever**	**1,031**	No	708	7	0.30	1,341	438	5	0.22
		Yes	292	24	2.65		866	62	1.40
									
**Vomiting**	**787**	No	714	24	1.00	1,380	1,212	63	1.02
		Yes	48	1	0.78		101	4	0.78
									
**Anyconvulsions**	**1,031**	No	937	24	0.81	1,380	1,213	42	0.68
		Yes	63	7	3.58		100	25	4. 90
									
**Partial convulsions**	**1,018**	No	984	15	0.92	1,380	1,286	59	0.90
		yes	16	3	6.05		27	8	5.81
									
**Able to breast feed**	**1,031**	No	407	24	1.31	1,372	55	9	1.92
		Yes	593	7	0.56		1,250	58	2.83
									
**Born in hospital**	**1,031**	No	632	25	1.28	1,373	1055	63	1.14
		yes	368	6	0.53		250	5	0.39

**Table 3:** Crude likelihood ratios for meningitis of items of clinical examination amongst neonates and young infants in the derivation period.

			First week of life				Age 7 to 59 days		
	Sample size		Without meningitis	With meningitis	Crude LR	Sample size	Without meningitis	With meningitis	Crude LR
**Neck stiffness**	**1,031**	**No**	995	31	1.01	1,380	1,285	60	0.92
		**Yes**	5	0	-		28	7	4.90
									
**Bulging fontanel**	**1,031**	**No**	990	26	0.85	1,380	1,300	56	0.84
		**Yes**	10	5	16.13		13	11	16.58
									
**Abnormal cry or none**	**1031**	**No**	949	30	1.02	1,380	1,287	65	0.99
		**Yes**	51	1	0.63		26	2	1.51
									
**Irritability**	**1031**	**No**	988	29	0.95	1,380	1,267	57	0.88
		**Yes**	12	2	5.38		46	10	4.26
									
**Spontaneous movements**	**1,031**	**No**	27	0	-	1,390	13	3	4.52
		**Yes**	973	31	1.07		1,300	64	0.97
									
**Drowsy, lethargic or unconscious**	**1,031**	**No**	782	21	0.87	1,380	1,153	51	0.87
		**Yes**	218	10	1.48		160	16	1.96
									
**Capillary refill seconds**	**1,031**	**< 3**	866	30	1.12	1,380	1,234	63	1.00
		**≥ 3**	134	1	0.24		79	4	0.99
									
**Weight(kg)**	**1,029**	**< 1.5**	115	0	-	1,370	36	0	-
		**1.5-1.99**	127	3	0.76		96	8	1.62
		**2-2.49**	195	6	0.99		152	12	1.54
		**≥ 2.5**	561	22	1.26		1,019	47	0.90
									
**Axillary temperature °C**	**1,031**	**< 36**	248	7	0.91	1,380	82	3	0.72
		**36-37.4**	546	8	0.47		752	24	0.63
		**37.5-38.9**	188	12	2.06		397	24	1.19
		**≥ 39**	18	4	7.17		82	16	3.82
									

When adjusted in a multivariate model, a history of fever, history of convulsions, irritability, bulging fontanel and an axillary temperature ≥ 39°C remained independent predictors of meningitis. Importantly, in the multivariate analysis, lack of a history of fever was strongly associated with the absence of meningitis. A simplified scoring system was constructed from these signs The minimum possible score was 0 with a maximum of 6 (table 4). 26/361 (7.2%) infants in the first week of life presenting with one or more of fever, convulsions, irritability, a bulging fontanel or an axillary temperature ≥ 39°C had meningitis compared to 5/670 (< 1%) without any of these signs (p < 0.001): sensitivity 84%, specificity 67%, PPV 7.2% and NPV 99.3%. Among infants in the first week of life with one or more of the signs of meningitis in young infants suggested by the current WHO guidelines, 24/509 (4.7%) had meningitis compared to 14/522 (2.7%) without any of these signs (P = 0.07).

**Table 4:** Multivariate adjusted likelihood ratios for indicators of meningitis amongst neonates and young infants in the derivation period with simplified scores.

Indicator		Adjusted Likelihood Ratios		Simplified scores	
		First Week of Life	Age 7 to 59 days	First week of life	Age 7 to 59 days
**History of Fever**	**No**	0.41	0.29	-1	-1
	**Yes**	2.16	1.32	1	0
					
**History of convulsions**	**No**	0.90	0.72	0	0
	**Yes**	2.03	3.89	1	1
					
**Bulging fontanel**	**No**	0.88	0.88	0	0
	**Yes**	8.46	8.56	2	2
					
**Irritability**	**No**	0.96	0.96	0	0
	**Yes**	3.83	1.91	1	1
					
**Axillary temperature °C**	**< 36**	0.94	0.78	0	0
	**36-37.4**	0.62	0.70	0	0
	**37.5-38.9**	1.59	1.14	0	0
	**≥ 39**	3.57	2.76	1	1
**constant**				+1	
**Composite total**				6	6

Amongst young infants outside the first week of life, 62/67 (93%) with meningitis had a history of fever. The absence of fever strongly predicted the absence of meningitis (table 3). Variables with a crude positive likelihood ratio ≥ 1.5 were convulsions, inability to feed, neck stiffness, bulging fontanel, irritability, absence of spontaneous movements, abnormal or absent cry and high axillary temperature. On multivariate analysis, history of fever, convulsions, bulging fontanel, agitation/irritability, and axillary temperature remained independent indicators of meningitis. Likewise these variables had a similar score (table 4). 63/986 (6.4%) infants age 7 to 59 days presenting with one or more of fever, convulsions, irritability, a bulging fontanel or an axillary temperature ≥ 39°C had meningitis compared to 4/394(1%) without any of these signs (p < 0.001): sensitivity 94%, specificity 30%, PPV 6.3% and NPV 99.0% for meningitis. Among infants, age 7 to 59 days with one or more of the WHO suggested signs of meningitis, 29/232 (12.5%) had meningitis compared to 38/1,140 (3.3%) without any of these signs (p < 0.001): sensitivity of 43%, a specificity of 84.4%, PPV 12.5% and NPV 96.6%.

Among both age groups, the scoring system using our signs, the best compromise on sensitivity and specificity was achieved with just a cut-off ≥ 1; ROC 0.79 (95%CI 0.75-0.84) sensitivity 90.8%, specificity 45.6% and did not differ between the first week of life or thereafter (p = 0.8). The WHO recommended signs had an ROC of 0.68 (95%CI 0.63-0.73) with a sensitivity 70.4%, specificity 61.9%.

### Validation

1,678 consecutively admitted neonates and young infants, of whom 900 (54%) were admitted in the first week of life and 778 (46%) aged 7-59 days were admitted during the validation period. Of these, 166 (10%) (141 during the first week of life and 25 thereafter) died without an LP and thus only 1,512 were included for validation. Overall 1,109 (66%) were born at home. During this period LPs were performed in 501/759 (66%) of neonates aged < 7 days and 532/753 (71%) of those aged 7 to 59 days. The overall proportion having an LP was greater in the validation period than in the derivation period (p < 0.001).

Meningitis was present in 63 (4.2%), 15/759 (2.0%) in the first week of life and 48/753 (6.4%) from 7-59 days. This prevalence of meningitis did not differ to from the derivation period either in the first week of life or 7-59 days (P = 0.45 & 0.13).

The overall area under the ROC curve for predicting meningitis with the indicators we had identified was 0.75 (95%CI 0.68-0.82). The area was (0.62(95%CI 0.49-0.75)) in the first week of life and (0.76(95%CI 0.68-0.85)) after the first week of life. This difference did not reach statistical significance (χ^2 ^= 3.24, P = 0.07). The overall area under the ROC curve for the signs suggested by WHO was 0.74(95%CI 0.67-0.81). It was significantly different (0.50(95%CI 0.35-0.65)) in the first week of life compared to (0.82(95%CI 0.75-0.89)) after the first week of life, (χ^2 ^= 14.69, P = 0.0001). The overall sensitivity using the signs we derived (78%), was not significantly different from the (70%) for the WHO signs. Likewise when applied the devised simplified scoring system to the validation data set, the best compromise on sensitivity and specificity was similarly achieved with just a cut-off ≥ 1; sensitivity 77.8%, specificity 55.2%.

Overall, one case of meningitis would be identified for every 21 (95% CI 15-35) infants who received an LP or presumptive treatment on the basis of the signs we found compared to 28 (95% CI 19-61) using the WHO recommended signs (Table 5).

**Table 5:** Overall performance of indicators of meningitis among neonates and young infants in the validation period

Indicators	Number with indicator	Number with meningitis	Sensitivity %	Specificity %	PPV %	NPV %	N.N.LP*	95% CI
Bulging fontanel	24	11	17.5 (9.5 to 25.4)	99.1 (98.7 to 99.5)	45.8 (25.0 to 67.0)	96.5 (96.2 to 96.8)	2	1 to 5

Convulsions or any of the above	128	31	49.2(34.9 to 66.7)	93.3(92.7 to 94.1)	24.2 (17.2 to 32.8)	97.7 (97.0 to 98.5)	5	3 to 7
Axillary temp ≥ 39°C or any of the above	187	34	54.0(38.1 to 73.0)	89.4 (88.8 to 90.3)	18.2(12.8 to 24.6)	97.8(97.1 to 98.7)	7	5 to 11
Agitation/irritability or any of the above	205	35	55.7(39.7 to 74.6)	88.3(87.6 to 89.1)	17.1(12.2 to 22.9)	97.9(97.1 to 98.7)	7	5 to 11
History of fever or any of the above	738	49	77.8(58.7 to 93.7)	52.5 (51.2 to 53.1)	6.6 (5.0 to 8.0)	98.2 (96.6 to 99.5)	21	15 to 35
One or more of the WHO suggested signs	636	44	69.8 (50.8 to 92.1)	59.1(58.3 to 60.1)	6.9 (5.0 to 9.1)	97.8 (96.5 to 99.4)	28	18 to 61
One or more of the WHO suggested signs plus history of fever	1,113	55	87.3(65.0 to 100)	27 (26.0 to 27.5)	4.9 (3.7 to 5.7)	98 (94.5 to 100)	42	24 to 154
All admissions	1,512	63	100	0	-	-	-	-

N.N.LP* Number of lumbar punctures needed to identify one case of Meningitis

## Discussion

Lumbar puncture with CSF microscopy, biochemical, and microbiological analysis is the only means of accurately diagnosing meningitis in young infants. We aimed to independently identify simple indicators of meningitis that could be used as a practical screening tool to detect infants that warrant a LP and to evaluate the signs suggested by WHO [3,12,13,16]. We also determined the feasibility of developing a simplified scoring system for diagnosing meningitis in neonates and young infants. We found that 'specific' signs of convulsion(s) and bulging fontanel were relatively specific, but insensitive. We found no evidence that the WHO 'general' signs of drowsiness, lethargy, unconsciousness, or reduced feeding were predictive of meningitis. Amongst clinical features not included in the WHO guidelines, we found a history of fever or a measured temperature ≥ 39°C to be useful indicators of meningitis. Importantly our finding that a cut-off of just ≥ 1 on a simplified scoring system offered the best compromise on sensitivity and specificity suggested that a scoring system may not be desirable, rather that any neonates or young infant with any one of the indicators indentified should be fully investigated for meningitis.

The sensitivity of our signs and the WHO signs was similar to that we previously found among older children [13], suggesting that meningitis may not be more difficult to identify in young infants. Importantly, our finding that 15 to 35 newborns and young infants need to be evaluated by LP to diagnose one case of meningitis highlights the burden the disease in our setting compared to a developed country where 30 to 90 newborns may need to be evaluated to diagnose one case [23]. Overall both our signs and those suggested by WHO had low specificity. However, given the seriousness of meningitis, and challenges in diagnosis and management especially in resource poor regions, clinical indicators with good sensitivity, which may encourage a low threshold for performing LP while retaining reasonable specificity are desirable.

The clinical indicators that we found did not differ from those found in infants aged 7-59 days, nor was there evidence that these clinical indicators performed differently in the first week of life. Our data suggest that LP or empirical treatment for meningitis should be done among infants in the first week of life who are sufficiently ill to require hospital admission and are found to have any one of the following clinical signs or complaints of a bulging fontanel, an history of convulsion(s), measured axillary temperature ≥ 39°C, agitation or irritable or report a history of fever. Infants with respiratory or circulatory compromise should receive parenteral antibiotics and an LP should be performed later to determine the duration of therapy. It is worth noting that the area under the ROC curve in the validation period using the WHO suggested signs did not significantly differ from chance (ROC area 0.5) for this age bracket. This suggests that the WHO signs may largely not be able to discriminate between those with or without meningitis in the first week of life.

In young infants' age between 7 and 59 days old, there were few cases of meningitis in the absence of a history of fever. The presence of one or more of four clinical features: a history of fever, a bulging fontanel, irritability, convulsions, or an axillary temperature ≥ 39°C identified 85% of the cases of meningitis at admission. The lower sensitivity of the signs suggested in the WHO guidelines [3] may reflect the fact that in 8/48 (17%) meningitis cases in the validation period, the only predictive clinical sign was a history of fever. It has previously been observed that rules requiring the presence of fever plus another sign result in loss of sensitivity [10], our results support this and suggest that neonates and young infants requiring admission and with either history of fever or measured axillary temperature ≥ 39°C should be evaluated by LP for meningitis.

We are encouraged that the clinical signs that we identified are contained within the set of 14 signs predictive of sepsis identified from data collected in the WHO multicenter study and were specifically associated with meningitis [8-10]. The principal differences were the inclusion of a history of fever and the exclusion of drowsiness, lethargy, coma or reduced feeding, which were not independently predictive.

Importantly, given the lack of microbiological laboratory resources at most hospitals in a rural African setting, clinicians may consider LP not worthwhile. However, we found that 90% of cases of meningitis in this age group are identifiable macroscopically or by basic CSF leukocyte counting. This underscores the value of undertaking LP in the absence of full biochemical or microbiological laboratory facilities.

### Limitations

We did not perform LPs in infants with compromised respiratory or circulatory status and many infants died before LP was undertaken. It is therefore likely that we missed meningitis in some of these cases, especially on the first day of life and amongst those with very low birth weight (although all sick newborns infants did receive appropriate intravenous broad-spectrum antibiotics for presumed sepsis as standard policy). We excluded these cases, but it may have reduced the predictive value of the signs we examined. Future studies should explore acceptability of post mortem LPs in resource poor nations, as this may aid in acquiring more robust data. Ajayi and Mokoulu reported from Nigeria that meningitis was rare in the first week of life. However in their study, three times fewer LPs were done in infants in the first 72 hours of life [24]. Secondly, our study lacked data on maternal and obstetric factors that might predict meningitis. However, the yield from performing LPs in infants in the first week of life with only maternal risk factors has previously been found to be low [25]. Thirdly, our findings are only from one site. It is thus plausible that the findings from this part of sub-Saharan Africa are not directly applicable to other areas of the world.

## Conclusions

In many hospitals in sub-Saharan Africa, neonates and young infants are initially assessed and treated by staff with little specific training or experience in neonatal or young infant care. Although clinical signs predictive of severe illness in this age bracket have been identified, previous studies did not specifically address meningitis [26]. Given that we found meningitis in only 1 in 21 investigated cases, building capacity and willingness to undertake LPs and perform basic CSF microscopy is critical. This may also be cost saving by avoiding unnecessary extended antibiotic treatment and inpatient stay in the majority of infants who do not have meningitis Importantly, continuous inpatient review is vital because one third of cases of meningitis are not recognised at admission. Any neonate or young infant with any one of the indicators indentified should be fully investigated. Where LP is initially contra-indicated, empirical treatment for meningitis should be started in newborns and young infants, and LP conducted as early as possible to determine the duration of antibiotic therapy.

## Competing interests

The authors declare that they have no competing interests.

## Authors' contributions

MKM provided inpatient neonatal care, data collection, conducted analysis and prepared the manuscript for submission. JAB conceived and designed the study, conducted the statistical analysis and the overall manuscript preparation. CRJCN was involved in the conception of the study, and with ME supervised clinical care and participated in analysis. AT participated in data collection and analysis. BSL was responsible for laboratory analysis and data, PJ and EW participated in patient care and data collection and manuscript preparation. All authors read and approved the final manuscript for publication. All members of the KEMRI medical, nursing, laboratory and computing team participated in patient care, data collection and data storage.

## Pre-publication history

The pre-publication history for this paper can be accessed here:

http://www.biomedcentral.com/1471-2334/11/301/prepub
